# Sequencing Multiple Cotton Genomes Reveals Complex Structures and Lays Foundation for Breeding

**DOI:** 10.3389/fpls.2020.560096

**Published:** 2020-09-16

**Authors:** Yuxin Pan, Fanbo Meng, Xiyin Wang

**Affiliations:** ^1^ Center for Genomics and Computational Biology, North China University of Science and Technology, Tangshan, China; ^2^ National Key Laboratory for North China Crop Improvement and Regulation, Agriculture University of Hebei, Baoding, China

**Keywords:** cotton, genome, phylogeny, transposable elements, fiber

## Abstract

Cotton is a major fiber plant, which provides raw materials for clothing, protecting humans from the harsh environment of cold or hot weathers, enriching the culture and custom of human societies. Due to its importance, the diploid and tetraploid genomes of different cotton plants have been repeatedly sequenced to obtain their complete and fine genome sequences. These valuable genome data sets revealed the evolutionary past of the cotton plants, which were recursively affected by polyploidization, with a decaploidization contributing to the formation of the genus *Gossypium*, and a neo-tetraploidization contributing to the formation of nowadays widely cultivated cotton plants. Post-polyploidization genome instability resulted in numerous structural changes of the genomes, such as gene loss, DNA inversion and translocation, illegitimate recombination, and accumulation of repetitive sequences, and functional innovation accompanied by elevated evolutionary rates of genes. Many these changes have been asymmetric between subgnomes of the tetraploid cottons, rendering their divergent profiles of biological regulation and function. The availability of whole-genome sequences has now paved the way to identify and clone functional genes, e.g., those relating to fiber development, and to enhance breeding efforts to cultivate cottons to produce high-yield and high-quality fibers, and to resist environmental and biological stress.

## Introduction

Cotton is the world economic crop for its natural textile fiber, averaging about 25% of total world fiber use. Cotton seeds are rich in oil and proteins, and therefore used for oil production and as feed supplement for cattle and sheep, or as raw materials to manufacture industrial products, such as soaps and cosmetics. More than 80 countries produce cotton, distributed in arid and semi-arid regions of the tropics and sub-tropics. The top three cotton producers are India, China, and the United States. World consumption of cotton fiber and seed oil was approximately 119.4 million bales and 43.67 million metric tons in 2018, respectively (U.S. Department of Agriculture, https://usda.library.cornell.edu/).

## Classification of Cotton Species

Cotton (*Gossypium* spp.) is an outstanding model species system for the plant cell elongation, cell wall, cellulose biosynthesis, and polyploidy research (Kim and Triplett, 2001). It is from Malvaceae family, including four subgenera, *G. subg. Gossypium*, *G. subg. Houzingenia*, *G. subg. Karpas,* and *G. subg. Sturtia*, and having approximately 45 diploid (2n = 2X = 26) and seven tetraploid (2n = 4X = 52) species (Hawkins et al., 2006; Salmon et al., 2010; Grover et al., 2015; Ditta et al., 2018). About 12.5 million years ago (Mya), *Gossypium* was diverged from its closer relatives *Kokia* and *Gossypioides* during the Miocene (Cronn et al., 2002).

Eight diploid cytogenetic genomes, designated as A to G and K, have been found in the world (Wang et al., 2012). The A, B, E, and F genomes occur naturally in Africa and Asia (Cronn et al., 2003). *G. herbaceum* (A_1_) is originated from Africa, and the primitive *G. arboreum* (A_2_) from India. D genome occurs in Americas, with *G. raimondii* (D_5_) initially found in Peru. The C, G, and K genomes are found in Australia (Chen et al., 2007). The haploid genome sizes vary from 2,500 Mb in the K genome, to about 800 Mb in the D genome (Hawkins et al., 2006).

**Figure 1:**
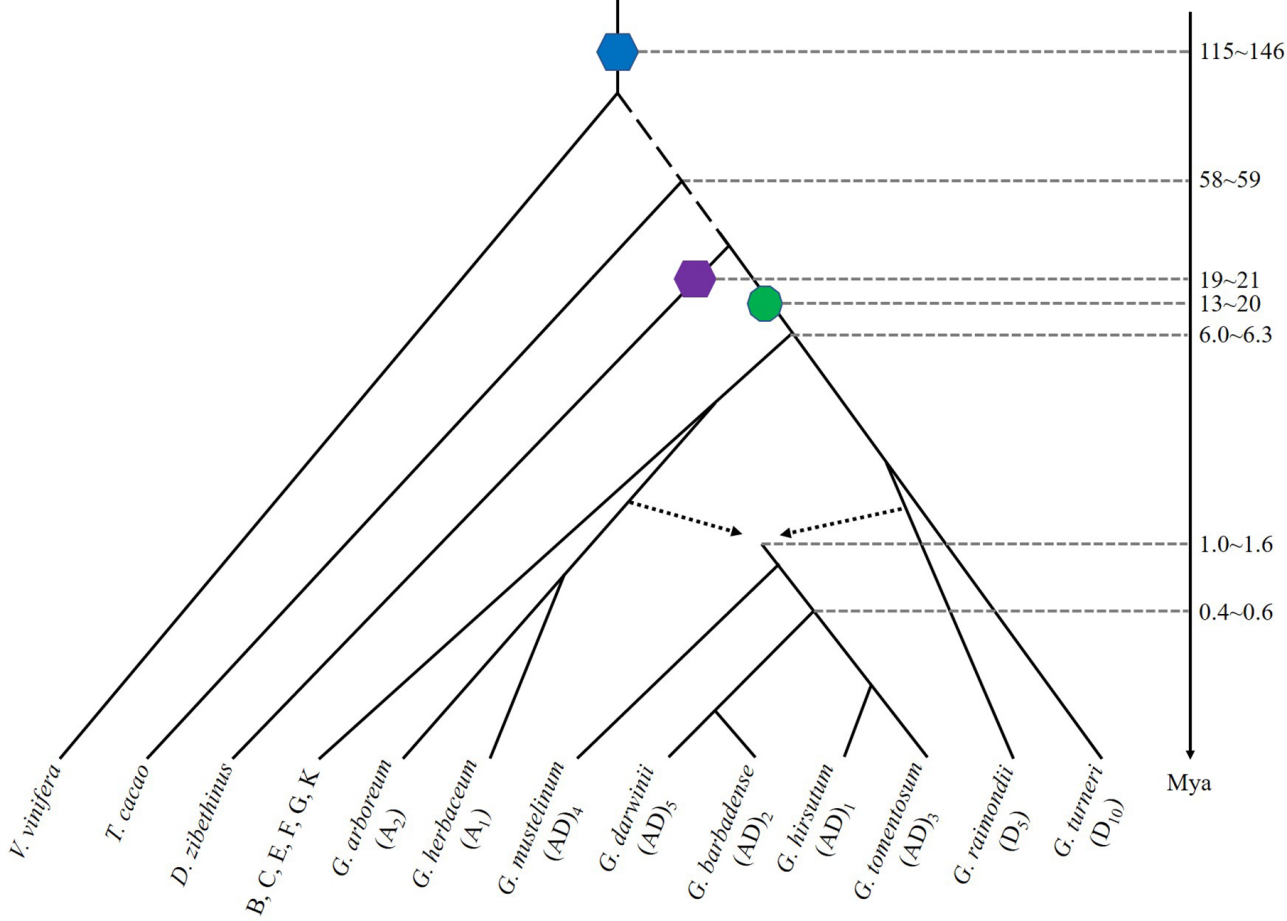
Evolutionary relationships of diploid and tetraploid species of *Gossypium* and related plants. The blue hexagon denotes a hexaploidization event shared by major eudicots, purple hexagon denotes a hexaploidization in Durian, and green decagon denotes a decaploidization shared by *Gossypium* species. Broken arrowed lines show a hybridization to produce the ancestral allotetraploid cotton. A time line is shown with approximate dates of key evolutionary events. Mya: millions years ago.

The A genome, African species much like modern *G. herbaceum* (A_1_) and *G. arboreum* (A_2_), and D genome like American diploid species, *G. raimondii* (D_5_), were reunited by trans-oceanic dispersal and chromosome doubling to give rise to allotetraploid cotton species (Paterson et al., 2012). Five widely-recognized tetraploid species include *G. hirsutum* (AD_1_), or “Upland cotton”, *G. barbadense* (AD_2_), “Pima” or “Egyptian” cotton, and three other exclusively wild polyploid species, *G. tomentosum* (AD_3_), *G. mustelinum* (AD_4_), and *G. darwinii* (AD_5_), are endemic to coastal and island habitats (Salmon et al., 2010). *G. hirsutum* (AD_1_) and *G. barbadense* (AD_2_), widely cultivated tetraploid cotton species, arose in the American continent, with the former in Mexico and the latter in Peru. These facts suggest that parallel and convergent domestication occurred (**Figure 1**).

### Cotton Genome Sequencing Efforts

In view of cotton’s importance in human life, intensive efforts have been focused on uncovering the genome mysteries of cotton species. Three diploid cottons and two tetraploid cottons were sequenced, including *G. arboreum* (Li et al., 2014; Du et al., 2018; Huang et al., 2020), *G. herbaceum* (Huang et al., 2020), *G. raimondii* (Wang et al., 2012; Paterson et al., 2012; Udall et al., 2019), *G. hirsutum* (Li et al., 2015; Zhang et al., 2015; Hu et al., 2019; Wang M. et al., 2019; Chen et al., 2020; Huang et al., 2020) and *G. barbadense* (Yuan et al., 2015; Liu et al., 2015; Hu et al., 2019; Wang M. et al., 2019; Chen et al., 2020). Detailed genome information is shown in **Tables 1** and **2**.

**Table 1:** Characteristics of *G. arboreum*, *G. herbaceum*, and *G. raimondii* genome sequences.

**Species**	***G. arboretum*(** Li et al., 2014 **)**	***G. arboretum*(** Du et al., 2018 **)**	***G. arboretum*(** Huang et al., 2020 **)**	***G. herbaceum*(** Huang et al., 2020 **)**	***G. raimondii*(** Wang et al., 2012 **)**	***G. raimondii*(** Paterson et al., 2012 **)**	***G. raimondii*(** Udall et al., 2019 **)**
Total contigs	40,381	8,223	2,432	1,781	41,307	19,735	187
Contig N50 (kb)	72	1,100	1,832	1,915	44.9	135.6	6,291
Total contig length (Mb)	1,561	1,710	1,637	1,556	744.4	748.1	734.9
Anchored contigs (Mb)	NA	1,573	1,509	1,489	NA	NA	NA
Total scaffolds	7,914	4,516	1,269	732	4,715	1,084	NA
Scaffold N50 (kb)	665.8	NA	NA	NA	2,284	18,800	58,819
Total scaffold length (Mb)	1,694	NA	NA	NA	775.2	761.4	734.9
Anchored and oriented scaffolds (Mb)	1,532	NA	NA	NA	406.3	748.7	NA
Total genes	41,330	40,960	43,278	43,952	40,976	NA	41,030

NA, not available.

**Table 2:** Characteristics of *G. barbadense* and *G. hirsutum* genome sequences.

**Species**	***G. hirsutum* (TM-1,V1.0)(** Li et al., 2015 **)**	***G. hirsutum* (TM-1,V1.1)(** Zhang et al., 2015 **)**	***G. hirsutum* (TM-1)(** Wang M. et al., 2019 **)**	***G. hirsutum* (TM-1,V2.1)(** Hu et al., 2019 **)**	***G. hirsutum*(TM-1,updated V1)(** Huang et al., 2020 **)**	***G. hirsutum*(TM-1)(** Chen et al., 2020 **)**	***G. barbadense* (Xinhai21)(** Liu et al., 2015 **)**	***G. barbadense* (3-79)(** Yuan et al., 2015 **)**	***G. barbadense* (3-79)(** Wang M. et al., 2019 **)**	***G. barbadense* (Hai7124,V1.1)(** Hu et al., 2019 **)**	***G. barbadense* (3-79,V1.1)(** Chen et al., 2020 **)**
Scaffold number	8,591	40,407	2,190	48	342	1,025	NA	29,751	3,032	11,701	4,748
Scaffold length (Mb)	2,173	2,432.7	2,347	2,295.3	2,290	2,305.2	NA	2,573.2	2,266.7	2,224.98	2,195.8
Scaffold N50 (Mb)	0.764	1.6	97.8	15.5	NA	108.1	0.503	0.26	92.9	23.44	93.8
Contig number	44,816	265,279	4,746	NA	1,235	6,733	NA	NA	4,930	75,898	4,767
Contig length (Mb)	2,090.4	2,068.1	2,281.9	2,267.9	NA	2,302.3	NA	NA	2,222.5	2,192.5	2,193.9
Contig N50 (kb)	80.38	34.0	1,891.9	113.3	5,020	783.9	72	NA	2,151	77.66	1,769.6
Total gene	76,943	70,478	70,199	72,761	74,350	75,376	77,526	80,876	71,297	75,071	74,561
Transposable element (Mb)	1,445.06	1,339	1,640	1,460.1	1,467.55	NA	1,391.48	1,778.62	1,582.8	1,374.61	NA

NA, not available.

Having relatively smaller sizes, the diploid genomes were firstly sequenced to provide a reference to explore the tetraploid genomes. Using Illumina HiSeq 2000 platform, the genome of *G. raimondii* was deciphered and assembled into 775.2 Mb length (Wang et al., 2012). At the meantime, by using Sanger, Roche 454 XLR and Illumina Genome Analyzer IIx platform, another *G. raimondii* genome was** sequenced, and based on the cotton genetic and physical maps, 761.4 Mb data was assembled and oriented to 98.3% of the expected genome size (Paterson et al., 2012)*.* Seven years later,** deciphered using PacBio long-read technology, HiC, and Bionano optical mapping, the third genome sequence of *G. raimondii* was published (Udall et al., 2019). In this assembled genome, the number of contigs decreased and the length of contig N50 was much elongated (6.3 Mb). The A genome from *G. arboreum* has a genome size almost twice of that of the D genome from *G. raimondii*. Using Illumina HiSeq 2000 platform, *G. arboreum* genome was sequenced and a total of 1,694 Mb was assembled with 90% oriented onto 13 pseudochromosomes (Li et al., 2014). In 2018, using PacBio and Hi-C technology, *G. arboreum* genome was re-sequenced again, and a total of 1,710 Mb contigs was assembled and 92% of contigs oriented (Du et al., 2018). Very recently, an improved genome sequence of *G. arboreum* with 1,637 Mb assembled DNA was published (with 92% oriented) (Huang et al., 2020). *G.herbaceum* var. *africanum,* another A-genome diploid cotton, was sequenced using Illumina, PacBio and Hi-C sequence technology. The assembled size was 1,556 Mb with 95.69% (1,489 Mb) of all sequenced oriented and organized into 13 chromosomes (Huang et al., 2020).

*G. hirsutum,* known for its high lint production than any other cultivated cotton species, accounts for more than 90% of commercial cotton production worldwide (Cronn et al., 2003). Acc. Texas Marker-1 (TM-1) is a genetic standard for *G. hirsutum* genome. Using Ilumina and BAC sequencing, *G. hirsutum* genome was sequenced to produce a total of 2,173 Mb assembled sequence (V1.0), with 88.5% anchored and oriented to 26 pseudochromosomes. The anchored A_t_-subgenome was 1,170 Mb with 35,056 genes, whereas the D_t_-subgenome was 753 Mb with 37,086 genes (Li et al., 2015). By integrating Illumina, Sanger-sequenced BAC-end sequences, the assembled TM-1 genome sequence (V1.0), and an ultra-dense genetic map, a 2,432.7 Mb TM-1 genome sequence (V1.1) was produced. In the assembly V1.1, 218 misassembled scaffolds (442.2 Mb) in the assembly V1.0 was corrected (Zhang et al., 2015). Very recently, by using PacBio, Illumina HiSeq and Hi-C, a much improved *G. hirsutum* genome sequence was published (Huang et al., 2020).

*G. barbadense* (Pima cotton) is famous for its extra-long, strong and fine fiber, and its accessions 3–79 were sequenced, being annotated 80,876 protein-coding genes in total. The subgenome A_t_ (1.50 Gb) was found to have a double size as to the D_t_ (853 Mb) (Yuan et al., 2015). And also, researchers sequenced *G. barbadense* cv. Xinhai21, through using three next-generation sequencing platforms, Roche 454, Illumina Hiseq2000, and PacBio SMRT (Liu et al., 2015). This genome sequence covered 1.395 Gb of the subgenome A_t_ and 0.776 Gb of the D_t_.

The draft genome sequences of allotetraploid cotton species had been highly fragmented and incomplete. Especially, telomere, centromere, and repeat-rich regions were often poorly assembled. Therefore, allotetraploid cottons, *G. hirsutum* accession TM-1, and *G. barbadense* accession 3–79, were re-sequenced and annotated by integrating single-molecule real-time sequencing, BioNano optical mapping, and high-throughput chromosome conformation capture techniques (Hi-C). These re-sequencing efforts revealed 70,199 genes in *G. hirsutum* and 71,297 genes in *G. barbadense* (Wang M. et al., 2019). At the meantime, *G. hirsutum* accession TM-1 and *G. barbadense* accession Hai7124 were also re-sequenced and assembled by integrating non-PCR-based short-read sequencing, long-read-based gap closure, scaffolding, and orientation based on 3D proximity information derived from Hi-C data and from optical and genetic maps. 72,761 and 75,071 protein-coding genes were identified in TM-1 and Hai7124 (V1.1), respectively. Contiguity and completeness for regions with high content of repeats were improved, especially with centromeric regions for each chromosome (Hu et al., 2019). Very recently, based on single-molecule real-time, Illumina and Hi-C, another five allotetraploid cotton genomes were sequenced. The assembled genomes range in size from 2.2 to 2.3 Gb (Chen et al., 2020).

Each assembly genome has its advantages and significance. The newly (re)sequenced and assembled genomes, *G. arboreum, G. herbaceum, G. hirsutum* (Huang et al., 2020), *G. raimondii* (Udall et al., 2019), and *G. barbadense* (Wang M. et al., 2019) provided chromosome-scale references. Two prominent databases were set up to store these genome data sets, CottonGen (https://www.cottongen.org/) (Yu et al., 2014) and CottonFGD (https://cottonfgd.org/) (Zhu et al., 2017).

Based on the assembling information, we compared the gene pairs between the same species of the relatively newly sequenced genomes using Blastn (sequence identity > 99% and aligned length >80% of each gene) (**Table 3**). As to the two compared D genome sequences, the number of common genes is only 11,541, accounting up to about 1/3 of the total annotated genes in each genome. A comparison of two A genome sequences revealed ~3/5 of commonly annotated genes. Comparisons of tetraploid cottons also found prominent fractions of non-common or specific genes. These comparisons alert a careful use of the gene annotation, in case the same gene was not well decoded, with its full length, or exon/intron composition. Besides, a pan-genus re-annotation of cotton genes integrating *de novo* prediction software and homology searching, supported by complete transcriptome information, is indispensable to assist further biological and agricultural exploration. We also inferred orthologous genes (23,906–28,334 between diploid cottons and 44,672–62,708 between tetraploid cottons), which also implies much divergence in gene composition among different cottons (**Table 4**).

**Table 3:** Comparison of annotated genes in the updated or newly sequenced cotton genomes.

**Species 1**	**Specific genes**	**Species 2**	**Specific genes**	**Common genes**
*G. arboretum* (Du et al., 2018)	16,918	*G. arboretum* (Huang et al., 2020)	19,236	24,042
*G. raimondii* (Paterson et al., 2012)	25,964	*G. raimondii* (Udall et al., 2019)	29,202	11,541
*G. barbadense* (Chen et al., 2020)	6,504	*G. barbadense* (Wang M. et al., 2019)	3,240	68,057
*G. barbadense* (Hu et al., 2019)	27,308	*G. barbadense* (Wang M. et al., 2019)	23,534	47,763
*G. barbadense* (Hu et al., 2019)	25,083	*G. barbadense* (Chen et al., 2020)	24,573	49,988
*G. hirsutum* (Huang et al., 2020)	35,016	*G. hirsutum* (Chen et al., 2020)	36,042	39,334

**Table 4:** Orthologous genes between diploid or tetraploid cotton genomes.

**Species 1**	**Species 2**	**Orthologous**
*G. arboretum* (Huang et al., 2020)	*G. raimondii* (Udall et al., 2019)	23,906
*G. arboretum* (Huang et al., 2020)	*G. raimondii* (Paterson et al., 2012)	26,800
*G. arboretum* (Du et al., 2018)	*G. raimondii* (Udall et al., 2019)	24,503
*G. arboretum* (Du et al., 2018)	*G. raimondii* (Paterson et al., 2012)	28,334
*G. barbadense* (Wang M. et al., 2019)	*G. hirsutum* (Chen et al., 2020)	53,799
*G. barbadense* (Wang M. et al., 2019)	*G. hirsutum* (Huang et al., 2020)	45,020
*G. barbadense* (Chen et al., 2020)	*G. hirsutum* (Chen et al., 2020)	62,708
*G. barbadense* (Chen et al., 2020)	*G. hirsutum* (Huang et al., 2020)	44,672
*G. barbadense* (Hu et al., 2019)	*G. hirsutum* (Chen et al., 2020)	48,087
*G. barbadense* (Hu et al., 2019)	*G.hirsutum* (Huang et al., 2020)	45,434

## Polyploidization and Cotton Origination

Polyploidy is a significant evolutionary drive force in plants (Bowers et al., 2003; Tang et al., 2008a). Cotton is ideal for investigating polyploidy for having been affected by recursive polyploidization.

*Gossypium* shared an ancient hexaploidization event with the other core eudicots (Tang et al., 2008b). Shortly after divergence from cacao, the *Gossypium* lineage experienced multiple-fold ploidy increase (Paterson et al., 2012). Through detecting gene collinearity, five-times of duplicated regions in cotton to those in cacao and grape suggested a paleo-decaploidy, or penta-plication of the ancestral genome, implying a rather complex nature of the cotton genome, as compared to many other eudicot plants (Wang et al., 2016). The obscurity whether cotton and the other Malvaceae plants share the event were discussed by comparing genomic information from durian and *Bombax* (Teh et al., 2017; Conover et al., 2019). Exploration of the collinear genes, including inter-genomic ratio of retained homologs, homologous gene tree topology, and gene retention levels suggested that the above-mentioned decaploidy was not shared with durian, which was affected by an independent hexaploidization (Wang J. et al., 2019). Actually, the decaploidy might have directly contributed to the origination and divergence of the *Gossypium* plants. Besides, it might continue to drive their evolution and functional innovation in that much increased mutations were observed in the duplicated genes, resulting in aberrant topology of gene trees (Wang J. et al., 2019). At least 83.2% of phylogenetic trees constructed with collinear homologs, including grape, cotton, and cacao, did not conform to the expected topology, clearly due to elevated evolutionary rates of certain duplicated gene copies (Meng et al., 2020).

However, it has been controversial about the time of cotton origination and speciation. Molecular phylogenetic analyses suggested that the common ancestor of *G. arboreum* and *G. raimondii* was diverged from *T. cacao* around 18–58 Mya (Li et al., 2014). By examining 745 single-copy gene families from nine sequenced plant genomes, *G. raimondii* and *T. cacao* were inferred to have** probably been diverged approximately 33.7 Mya from a common ancestor (Wang et al., 2012). A latest estimation suggested that the divergence between *Gossypium* and *T. cacao* occurred about 58−59 Mya (Chen et al., 2020). Using synonymous substitution rates (Ks) obtained from 3,195 paralogous gene pairs in the *G. raimondii* and *T. cacao* genomes, two Ks value peaks were observed at 0.40–0.60 and 1.5–1.90. Corresponding to the peak with smaller Ks, the decaploidization event was proposed to occurred approximately 16.6 (13.3–20.0) Mya in the *Gossypium* lineages. The second peak corresponds to the more ancient hexaploidization event shared by core eudicots, having occurred approximately 130.8 (115.4–146.1) Mya (Tang et al., 2008b; Van de Peer et al., 2009). In *G. arboreum*, two coincident polyploidization events shared by *G. raimondii* were estimated using Ks of 1,917 paralogous gene pairs of similar age, with one peak around 0.17 synonymous transversions per site and a second peak at about 0.54 was observed.** This information showed the two polyploidization events** to have occurred at 13–20 and 115–146 Mya (Li et al., 2014). In a comparative analysis of cotton to cacao and durian, the cotton decaploidization was inferred to have occurred ~13–14 Mya and split from cacao ~21–24 Mya (Wang J. et al., 2019). Homeologous exchanges had occurred throughout the polyploid divergence and speciation in cotton (Salmon et al., 2010), possibly resulting in gene conversion, which made it even more complex to perform a reasonable evolutionary dating with gene or protein sequences (Guo et al., 2014).

All diploid cotton species retain 13 common chromosomes and largely collinear gene order. The A-genome and D-genome diploids were inferred to share a common ancestor about 5–10 Mya (Wendel and Albert, 1992; Argout et al., 2011; Wang et al., 2012). A most recent estimation of divergence time between the A and D was inferred to be 6.0–6.3 Mya (Zhang et al., 2015), or 6.2–7.1 Mya (Hu et al., 2019). A more recent estimation of 4.7–5.2 Mya was recently obtained (Chen et al., 2020). The common ancestor of the A_1_ and A_2_ clade was phylogenetically a sister to the A_t_- subgenomes of (AD)_1_ and of (AD)_2_, and the divergence time for A_1_ and A_2_ was estimated to be ~0.7 Mya, suggesting that allotetraploid formation (~1.0−1.6 Mya) preceded the speciation of A_1_ and A_2_ (Huang et al., 2020) (**Figure 1**).

Gene collinearity analysis between *G. hirsutum* and *G. barbadense* genome suggested that both were originated from a common allotetraploid ancestor (**Figure 1**). The hybridization between A- and D- genomes was initially inferred to occur 1 to 2 Mya (Fawcett et al., 2009). By comparing the sequenced *G. hirsutum*, *G. arboreum*, and *G. raimondii* orthologous genes, the ancestral allotetraploid was estimated to form about 1–1.5 Mya (Li et al., 2015). The divergence of the two allotetraploid cottons was inferred to occur ~1 Mya (Liu et al., 2015). An updated analysis with allotetraploid genomes inferred the tetraploidization to occur ~1.7–1.9 Mya and the divergence of *G. barbadense* and *G. hirsutum* was 0.4–0.6 Mya (corresponding to Ks peaks at 0.002 and 0.003, respectively) (Hu et al., 2019). Recently, the allotetraploid formation was estimated to ~1.0–1.6 Mya (Huang et al., 2020) and five allotetraploid genome sequences further illustrated the divergence between tetraploid and diploid clades occurred about the same time (Chen et al., 2020).

## Chromosomal Structural Conservative and Variations

Polyploidization may have impact on the architecture of the genome and structural changes may lead to phenotypic variation. Actually, 780 collinear blocks were detected between *G. raimondii* [JGI, (Paterson et al., 2012)] and *G. arboreum*. Based on inferred gene collinearity between orthologous chromosomes, large-scale rearrangements were inferred on chromosomes 2 and 3 of *G. raimondii*, while deletions and insertions were found on chromosomes 7 and 8 of *G. arboreum* (Li et al., 2014). With a comparative analysis of A_1_ (*G. herbaceum*) and A_2_ (*G. arboreum*) genomes, a reciprocal translocation was revealed on each of chromosomes 1 and 2, and two large-scale inversions were detected in chromosomes 10 (~18.4 to ~61.3 Mb in A_1_ and ~18.79 to ~58.96 Mb in A_2_) and 12 (~15.96 Mb to ~77.61 Mb in A_1_ and 15.66 to 84.69Mb in A_2_) of A_1_- and A_2_-genomes (Huang et al., 2020).

Owing to high gene collinearity between *G. hirsutum* and *G. barbadense* and that of each with their diploid progenitors or within themselves, DNA translocation and inversion were inferred in these genomes (Zhang et al., 2015; Huang et al., 2020). The A_t_ and D_t_ subgenomes had similar number of rearrangements (19 versus 18) (Zhang et al., 2015). Whereas, the Hi-C data indicates that chromosome rearrangements occurred in all 13 chromosomes between *G. babadense* and *G. hirsutum* genome sequences. A total of 170.2 Mb inverted DNA regions were identified and the majority were shared by both A_t_- subgenomes of *G. hirsutum* and *G. barbadense*. Four chromosomes exhibited paracentric inversions and eleven chromosomes showed pericentric inversions in heterochromatin. There were four large inversions, including three paracentric inversions and one pericentric inversion in the A06 chromosome between *G. hirsutum* and *G. barbadense* (Wang M. et al., 2019). Compared to *G. raimondii* (JGI, (Paterson et al., 2012), unique and shared structural variants were detected in *G. hirsutum* and *G, barbadense*, such as one large inversion in *G. barbadense* chromosome D05 and *G. hirsutum* D12, and a shared large inversion in the D09 chromosome was shared by both tetraploids. A large pericentric inversion was revealed in *G. barbadense* D05 (41.48–51.18 Mb) and *G. hirsutum* D12 (14.15–31.54 Mb), and a large inversion in the D09 was shared by both tetraploids (Wang M. et al., 2019). The A_1_- and A_2_-genomes were found to have two and three translocations, respectively, as compared to the A_t1_ subgenome (*G. hirsutum*), which is between chromosomes 2 and 3, and 4 and 5 in the tetraploid A_t1_ subgenome. An inversion in chromosome 10 occurred between A_1_ and A_t1_ (~18.4 Mb to ~61.3 Mb in A_1_ and ~23.09 Mb to ~97.42 Mb in A_t1_) (Huang et al., 2020). The D_t_-subgenomes had fewer and smaller inversions than the A_t_-subgenomes among five tetraploid cotton genomes, except for a few small inversions in D10 of Gt–Gm and Gm–Gb and D12 of Gd–Gt–Gm (Chen et al., 2020). Using the D_5_ genome [JGI, (Paterson et al., 2012)] as a reference, 77 putative translocation sites were observed in the 13 chromosomes of D_t_ and chromosomal translocations occurred more frequently in sub-telomeric regions in *G. barbadense* (Yuan et al., 2015). In the *G. hirsutum*, at least nine translocations and 28 inversions were identified. Two large reciprocal translocations were found between A02 and A03 and between A04 and A05, and three inversions found on the A12 and D12 homoeologous chromosomes (Zhang et al., 2015). Between *G. babadense* and *G. hirsutum* genome sequence, there were 3,820 translocations revealed (Wang M. et al., 2019). Also, there were translocations along chromosomes 1–3 and chromosomes 4 and 5 in *G. hirsutum* and *G. barbadense* (Hu et al., 2019).

### Expansion of Transposable Elements

Transposable elements (TEs) have important roles in driving genome evolution (Galindo-Gonzalez et al., 2017). They account up to 57% (441Mb) of the *G. raimondii* genome, mainly the *gypsy* and *copia*-like long terminal repeat (LTR), a type of retrotransposons, which explains much of the expansion of the *G. raimondii* genome. Data analysis indicated that the *G. arboreum* genome tended to harbor more LTRs inserted than *G. raimondii* genome during the last 0.5 million years (Cheng et al., 2019). *G. arboreum* genome had the greatest amount of repeat-containing sequences among sequenced cottons, and LTRs accounted for 95.12% of all repeat sequences (Cheng et al., 2019). In comparison to the *G. raimondii* genome, the *G. arboreum* genome had noticeable proliferation of *Gorge* elements. LTR retrotransposons in *G. arboreum* appeared to cluster near centromeres. Thus, LTR expansion seemed to contribute after a two-fold increase in *G. arboreum* genome (Li et al., 2014).

TEs of the D_t_-subgenome tend to be more active than those of the A_t_-subgenome after the tetraploidization. Both *copia* and *gypsy* were more actively transcribed in the D_t_-subgenome. *Copia* elements were remarkably more active than *gypsy* in the recent 0~1 My time, with higher proportions of *copia* located near coding genes than *gypsy* (Li et al., 2015). Two different methods inferred similar TE proportion in its genome (1,339 Mb versus 1,445 Mb, 64.8 versus 66%) (Li et al., 2015; Zhang et al., 2015). There were more TEs in the A_t_-subgenome (at least 843.5 Mb) than in the D_t_-subgenome (at least 433 Mb). Among them, the number of *gypsy* retro elements was 3-fold higher in the A_t_-subgenome than in the D_t_-subgenome. The TE types and relative proportions of A_t_- and D_t_-subgenome were similar to their corresponding genomes of *G. arboreum* and *G. raimondii*, whereas the retrotransposon frequencies were different (52.29 versus 62.81%). The TE divergence time was inferred to be older than 1.5 My, suggesting that most TEs expanded before the formation of allotetraploid cotton (Zhang et al., 2015). Moreover, TE expansion occurred in the progenitor genomes and was retained after allotetraploid formation. *Copia* elements had been remarkably more active than *gypsy*, with *copia* more frequently located near coding genes than *gyp*s*y*. Some *copia*- and *gypsy*-like elements were observed to be present in the D_t_ while absent in the D-genome (Chen et al., 2020). One of the two *G. barbadense* genomes were observed to have 83.5 and 82.2% LTRs in A_t_ and D_t_ (Liu et al., 2015), and the other detected 1,778.6 Mb of TEs (69.1% of the assembly), including 1,098.0 Mb of TE sequences in A_t_ (representing 73.5% of the subgenome) and 541.6 Mb of TEs in D_t_ (representing 63.5% of the subgenome) (Yuan et al., 2015). The latest data showed that A_t_ had 4.0–5.9% lower repetitive DNA content than the A-genome, whereas the D_t_ had 1.5–2.9% higher content than the D-genome. These changes may affect the downsizing and equilibration in allotetraploids (Chen et al., 2020).

A large number (9.15%) of LTR bursted at 5 Mya and decreased thereafter in A_t_, whereas a substantially lower and flat peak appeared 3−5 Mya in D_t_ (Liu et al., 2015). Whereas, the timing of insertion for LTRs was peaked within 1.9 Mya in D_t_ and approximately 3.1 Mya ago in A_t_ (Yuan et al., 2015). The above research suggested that the most expansions of extant LTRs independently occurred after the lineage separation but before tetraploidization (Wu et al., 2017). The two-fold expansion of A_1_ and A_2_-genomes and the *G. hirsutum* A_t1_-subgenome was found to be highly correlated to TE bursts. LTR families in *Gossypium* had been greatly expanded in comparison to durian [52.42% of the D_t1_-subgenome (*G. hirsutum*), 53.2% of the D_5_-genome, 26.2% of durian]. More than 72% genome is LTRs in A-genome (72.57% of the A_1_-genome and 73.62% of the A_2_-genome). LTR retrotransposons in *Gossypium* experienced continuing and more recent amplification bursts occurred about 0–2 Mya. Using the representative LTR/Gypsy sequences, TE burst time was evaluated to be related to cotton genome divergence. The earliest burst occurred ~5.7 Mya, which coincided the expected speciation time for A- and D- genome. The next one occurred ~2.0 Mya, observed specifically in D_t1_- and A_t1_-genomes (*G. hirsutum*), coinciding the formation of allotetraploid cotton (Huang et al., 2020).

## Asymmetric Evolution of the A and D Genome

The A genome species produce spinnable fiber, whereas the D genome species do not (Applequist et al., 2001), showing divergent contribution to phynotypes. Actually, with genome sequences, asymmetric evolution was found to have characterized the difference between genomes, including the above-mentioned difference in TE composition and expansion.

In *G. hirsutum*, the D_t_-subgenome had higher mutation rates than the A_t_-subgenome. The single-nucleotide variation rate in D_t_ versus D was greater than that in A_t_ versus A in intergenic collinear regions. Further analysis revealed that A- and A_t_-genomes had been undergoing greater positive selection than the D- and D_t_-genomes (Li et al., 2015). For example, the average Ks for collinearity-supported gene pairs in A_t_-and D_t_-subgenomes were found to be substantially lower than those of the A and D diploid genomes, respectively. In addition, D_t_/D versus *T. cacao* had lower dN/dS ratios than A_t_/A versus *T. cacao*. These data suggested that the genetic redundancy generated by allotetraploidy may have allowed relaxed purifying selection in both the A_t_- and D_t_-subgenomes (Li et al., 2015). There were similar levels of rearrangements between chromosomes and those between the A_t_- and D_t_-subgenomes (19 versus 18). However, the length of total rearrangements was revealed to be larger in the A_t_-subgenome (372.6 Mb) than in the D_t_-subgenome (82.6 Mb). The SNP frequency between *G. hirsutum* and *G. barbadense* was slightly larger in the A_t_-subgenome with 8,131,276 (5.95 per kb), than that in the D_t_-subgenome, with 4,685,422 (5.81 per kb) (Wang M. et al., 2019). All above information revealed that A_t_-subgenome had experienced more relaxed selection pressure (Yuan et al., 2015; Liu et al., 2015). In the same way, allele-SNPs within subgenomes, between accessions of six cotton tetraploid species, were detected to be higher in the A_t_-than D_t_-genomes. In both AD_1_ and AD_2_, the number of A_t_-subgenome allele-SNPs was observed about 1.5X that of D_t_-genome allele-SNPs (Page et al., 2016).

A systematic characterization of presence/absence variations (PAVs) between the two tetraploid accessions was also performed. With the PAVs, a lot of genes unique to *G. barbadense* were highly expressed during fiber development. Besides, there were also inversions between *G. hirsutum* and *G. barbadense*, including 120.4 Mb of A_t_-subgenome and 49.8 Mb of D_t_. By using Hi-C, large inversions were shown to have paracentric and pericentric inversions in the chromosomes A06 and D12 (Wang M. et al., 2019). Besides, introgressions were detected in AD_1_ and AD_2_ genomes owing to the attempts of breeders for transfer of genes for disease resistance, fiber quality, and other traits between (AD)_1_ and (AD)_2_ (Page et al., 2016).

Asymmetric DNA duplication phenomenon in tetraploid subgenomes was discovered through comparing accessions of *G. hirsutum and G. barbadense*. A_t_ DNA duplications were more (2X) conserved than D_t_ DNA duplications in *G. hirsutum* cultivars, although not in *G. barbadense*. Besides, A_t_ DNA deletions were more conserved than D_t_ DNA deletions in *G. barbadense* but not in *G. hirsutum*. Both these findings implied the existence of independent domestication events for these two species (Page et al., 2016).

Asymmetric changes in A_t_- and D_t_-subgenomes were also reflected in biased A_t_- or D_t_-homeolog expression (Yoo and Wendel, 2014). For example, more transcription factor genes (such as *MYB* family members) were expressed in the A_t_ homeologs, suggesting their important roles in fiber development. This might have led to subfunctionalization of the A_t_-D_t_ paralogs (Li et al., 2015).

Unidirectional DNA exchanges were the predominant mechanism responsible for allelic differences between the *Gossypium* tetraploids and their diploid progenitors. A_t_-to-D_t_ conversion was enriched in heterochromatin, which was highly correlated with GC content and transposon distribution, and may silence abundant A-genome-derived retrotransposons (Guo et al., 2014).

## Genomic Insight for Cotton Fiber Gene Expression and Development

Cotton fibers, which are highly elongated epidermal cells, undergoing four over-lapping development, initiation, elongation (Primary cell wall, PCW), secondary cell wall (SCW) synthesis and maturation (Basra and Malik, 1984). The elongation and SCW synthesis stages are key stages for fiber length and strength. At SCW stage, cross-linking of cellulose microfibrils and non-cellulosic matrices presumably “fix” the structure of the PCW, resulting in the first significant increase in fiber strength (Wilkins and Arpat, 2005).

Different development features in *Gossypium* genus may lead to typical fiber traits. *G. barbadense*, Hai7124, had an extended time longer than *G. hirsutum*, TM-1, in fiber elongation period. The stage lasted about 15 days from 5 to 25 days post-anthesis (DPA) in TM-1, whereas this lasted from 5 to 30 DPA in Hai7124. During the PCW-SCW transition period, expression of most genes is earlier in TM-1 than in Hai7124 (Zhu et al., 2011). Also, there are amount of expression data revealing the difference in the regulatory pathway. For example, translation and ribosome biosynthesis pathway genes are enriched during fiber elongation in Upland cotton and during cellulose biosynthesis in Pima cotton (Chen et al., 2020).

Based on genome sequences, more and more genes with structure and qualitative transcript differences in fiber development were identified. Actually, fiber elongates more than 2000-fold after initiation, which is regulated by cell turgor. The plasmodesmata on/off switch, together with sucrose and potassium ions (K^+^) transporter is curial for fiber cells (Ruan et al., 2004). Several genes associate with membrane transport, transcription, and glycan biosynthesis and carbon metabolism are significantly expressed in Hai7124, as compared to TM-1 fibers. Furthermore, genes regulating Hai7124 fiber biosynthesis, such as those encoding the sucrose transporter (*GbTST1*), Na^+^/H^+^ antiporter (*GbNHX1*), aluminum-activated malate transporter (*GbALMT16*), the vacuole-localized vacuolar (GbVIN1) and plasmodesmata (PD) opening have a longer period of expression than the corresponding genes in TM-1 (Hu et al., 2019).

The fiber SCW is consisted of >94% cellulose(Wilkins and Arpat, 2005). Sucrose synthase gene (sus) expressed in the later stage of fiber development plays a role in cell wall cellulose (Suppression of sucrose synthase gene expression represses cotton fiber cell initiation, elongation, and seed development). Between *G. raimondii* and *G. hirsutum* cotton ovules at 3 DPA, three Sus genes (*SusB*, *Sus1* and *SusD*) are expressed at substantially higher levels in *G. hirsutum* than in *G. raimondii* (Wang et al., 2012). *Cellulose synthase A* (*CesA*), synthesis cellulose, acts a key role in the regulating of the secondary cell wall thickening. Through genome-wide analysis, 32 *CesA* and 64 cellulose synthase-like (CSL) genes were detected in *G. hirsutum*, and 37 *CesA* genes identified in *G. barbadense.* The *CesA* genes can be classified into two major groups with six branches. One group is expressed during primary cell wall development, whereas the other group is expressed during secondary cell wall biosynthesis. Genes encoding CesA, UGD, UGP and UER, important for cotton fiber growth, locate in the A_t_-subgenome, are expressed highly during either the primary or the secondary cell wall biosynthesis stages (Yuan et al., 2015; Li et al., 2015).

Transcription factors are important for cotton fiber development. MYB is one of the most abundant transcription factors in cotton, and play diverse roles during cotton growth and evolution (Salih et al., 2016). A total of 219 and 524 MYB genes were identified in the *G. raimondii* and *G. hirsutum* genome (Wang et al., 2012; Salih et al., 2016). In *G. hirsutum*, two groups of MYB genes are expressed. One exhibits low or undetectable expression levels and the other express higher during -1 to 10DPA. A large number of MYB genes are expressed more predominantly in *G. hirsutum* ovules than *G. raimondii.* Some of MYB MIXTA-like (GhMML) homeologs are also highly expressed during fiber initiation in *G. hirsutum*, but down-regulated in fiberless mutants. All the above information indicates that some MYB genes might be required for early fiber development. WRKY, another transcription factor, participates in fiber development. In total, 112 and 109 WRKY genes were identified in *G. raimondii* and *G. arboreum*. Many SNPs are distributed unequally in exon and intron regions in these genes (Ding et al., 2015).

Ethylene is a key signaling modulator of cotton fiber cell growth (Shi et al., 2006; Pang et al., 2010). Among these, 1-aminocyclopropane-1-carboxylic acid oxidase (ACO) is the last enzyme in the ethylene synthesis. High amounts of transcripts relating to ACO activities were discovered from *G. raimondii* at the 3-DPA stage. ACO3 and ACO1 are 500- and 1000-fold higher than *G. arboreum*, respectively. CaACO loss of MYB-binding sites may lead to lower transcripts in *G. arboreum*. Compared to *G. raimondii*, ACO expression and ethylene are both lower in *G. hirsutum*. The ACO expression peak and ethylene burst occurred in the later stage of *G. hirsutum*. Very high levels of ACO transcripts will give rise to ethylene burst and may force an early fiber senescence phenotype, whereas the inactivation of ACO in *G. arboreum* ovules may be responsible of the short-fiber phenotype in this species. A compromise ACO expression may be the power for fiber traits in *G. hirsutum*. Thus, ACO is suggestive of a major role for the plant hormone ethylene during early fiber cell development (Wang et al., 2012; Li et al., 2014).

A total of 591 PSGs (72.8%) genes with the codons subjected to positive selection, express during the fiber development. Many of the A-homeologous PSGs are enriched in the synthesis of ethylene and very-long-chain fatty acids, sucrose metabolism and beta-d-glucan biosynthetic pathway to produce UDP-glucose, whereas the D-homeologous PSGs are enriched in carbohydrate transport, response to superoxide and other abiotic stresses. These results suggest that allotetraploid cotton domestication is associated with intensive human selection for fiber yield and quality on the A homeologs from fiber-producing species and for wider adaptation on the D homeologs from non-poor species. These findings suggested that A_t_-subgenome have contributed more to fiber improvement (Zhang et al., 2015).

## Conclusion and Future Prospects

The availability of multiple cotton genomes shed precious light on their genome structures and evolution, especially genome instability after recursive rounds of paleo-polyploidization. They contributed to understanding genes regulating the formation and elongation of fibers.

In this era of information technology and big data, biology-related technology advances every day and data is increasing at a fast pace. The big data produced requires to be quickly and deeply analyzed to yield useful knowledge. In the future, we will have a lot of opportunity to elucidate the cotton genome and other omics data to clarify the biology of cotton, especially the regulation of fiber biosynthesis. We look forward to seeing the big genome data can be fully analyzed and then applied to agricultural practices to improve the cotton yield quality, contributing to humans’ harmonious life on the earth.

## Author Contributions

XW conceived and led the research. YP and FM performed the analysis or joined discussion. XW and YP wrote the paper.

## Funding 

We appreciate financial support from Hebei Province Science and Technology Support Program (14962905D to YP), and Department of Education of Hebei Province (Y2012025 to YP), the Ministry of Science and Technology of the People’s Republic of China (2016YFD0101001 to XW), National Natural Science Foundation of China (3117022 to XW), and Tangshan Key Laboratory Project to XW.

## Conflict of Interest

The authors declare that the research was conducted in the absence of any commercial or financial relationships that could be construed as a potential conflict of interest.
